# Exercise Improves Cognitive Responses to Psychological Stress through Enhancement of Epigenetic Mechanisms and Gene Expression in the Dentate Gyrus

**DOI:** 10.1371/journal.pone.0004330

**Published:** 2009-01-30

**Authors:** Andrew Collins, Louise E. Hill, Yalini Chandramohan, Daniel Whitcomb, Susanne K. Droste, Johannes M. H. M. Reul

**Affiliations:** Henry Wellcome Laboratories for Integrative Neuroscience and Endocrinology, University of Bristol, Bristol, United Kingdom; James Cook University, Australia

## Abstract

**Background:**

We have shown previously that exercise benefits stress resistance and stress coping capabilities. Furthermore, we reported recently that epigenetic changes related to gene transcription are involved in memory formation of stressful events. In view of the enhanced coping capabilities in exercised subjects we investigated epigenetic, gene expression and behavioral changes in 4-weeks voluntarily exercised rats.

**Methodology/Principal Findings:**

Exercised and control rats coped differently when exposed to a novel environment. Whereas the control rats explored the new cage for the complete 30-min period, exercised animals only did so during the first 15 min after which they returned to sleeping or resting behavior. Both groups of animals showed similar behavioral responses in the initial forced swim session. When re-tested 24 h later however the exercised rats showed significantly more immobility behavior and less struggling and swimming. If rats were killed at 2 h after novelty or the initial swim test, i.e. at the peak of histone H3 phospho-acetylation and c-Fos induction, then the exercised rats showed a significantly higher number of dentate granule neurons expressing the histone modifications and immediate-early gene induction.

**Conclusions/Significance:**

Thus, irrespective of the behavioral response in the novel cage or initial forced swim session, the impact of the event at the dentate gyrus level was greater in exercised rats than in control animals. Furthermore, in view of our concept that the neuronal response in the dentate gyrus after forced swimming is involved in memory formation of the stressful event, the observations in exercised rats of enhanced neuronal responses as well as higher immobility responses in the re-test are consistent with the reportedly improved cognitive performance in these animals. Thus, improved stress coping in exercised subjects seems to involve enhanced cognitive capabilities possibly resulting from distinct epigenetic mechanisms in dentate gyrus neurons.

## Introduction

It is now well established that regular physical exercise has a positive impact on a range of biological systems, including the brain [1]–[6]. The resulting antidepressant-like and anxiolytic effects have led to exercise being proposed as an effective co-treatment (i.e. in addition to drug and behavioral therapies) for anxious and depressed patients [7]–[10]. Our previous work has indicated that voluntarily exercised animals show improved stress-coping in the face of physically demanding or psychological challenges. These improved stress-coping responses and strategies surfaced as more pertinent, adaptive responses of the hypothalamic-pituitary-adrenocortical (HPA) axis [11]–[13], improved sleep quality and enhanced stress resistance of sleep/EEG profiles [14], and decreased anxiety-related behavior and impulsivity in voluntary exercised mice and rats [15] relative to sedentary control animals. It is thought that these physiological and behavioral changes may well be of relevance for the clinical effects of exercise in patients suffering from stress-related mental disorders [7]–[9], [16], [17]. However, currently the underlying mechanisms of these beneficial effects of exercise are still largely unknown. In the present study it was our aim to gain more insight into the neurobiological basis of the enhanced stress-coping capabilities shown by voluntarily exercised animals.

In particular, we investigated the role of epigenetic mechanisms in the brain involved in transcriptional activation in coordinating adaptive behavioral responses to stressful events. Epigenetic mechanisms comprise of post-translational modifications of DNA and histone proteins within the chromatin structure, such as the methylation of DNA, and the acetylation, methylation, phosphorylation and other modifications of the N-terminal tails of distinct histone molecules [18]. Specifically of interest is the phosphorylation of serine-10 (Ser10) combined with the acetylation of lysine-14 (Lys14) in the N-terminal tail of histone H3 (i.e. P(Ser10)-Ac(Lys14)-H3) as this modification is thought to be involved in the local opening of condensed chromatin, thereby allowing the transcriptional induction of specific, hitherto silent genes [19], [20]. We postulated that these specific chromatin modifications are involved in triggering gene expression responses required for physiological and functional adjustments in neurons involved in the cognitive processing of stressful events [21].

Indeed, we could show that psychologically stressful events such as forced swimming and exposure to a novel environment enhances the phosphorylation and phospho-acetylation of histone H3 in a distinct population of dentate gyrus granule neurons in the hippocampus [21]–[24]. The response to stress was transient, peaking at 1–2 hours and coincided with the induction of c-Fos specifically in these neurons [23], [24]. Previous *in vitro* work has indeed shown that phospho-acetylation of histone H3 of the promotor region of the c-Fos gene occurs at induction of this immediate-early gene [19]. Furthermore, we obtained evidence that the phospho-acetylation of histone H3 and the induction of c-Fos is brought about by at least two, concurrently acting signaling pathways being the glucocorticoid receptor (GR) and the NMDA-R/ERK/MSK pathway (NMDA-R, N-methyl-D-aspartate receptor; ERK, extracellular signal-regulated kinase; MSK, mitogen- and stress-activated kinase) [21]–[24]. The epigenetic and gene expression responses in the dentate gyrus are thought to be involved in learning to cope with stressful, traumatic events as we recently obtained substantial evidence that these mechanisms are required for the formation of memories of the events [21], [22], [24], [25].

Here we investigated whether changes in histone H3 phospho-acetylation and gene expression responses would be involved in the enhanced stress coping capabilities seen in exercised subjects. Therefore, we subjected exercised and control rats to novelty exposure and forced swimming and investigated changes in dentate gyrus histone H3 phospho-acetylation and c-Fos expression, and acute behavioral responses as well as memory formation of the event.

## Materials and Methods

### Animals

Male Sprague-Dawley rats (140–160 g; purchased from Harlan, (Oxon, UK) were singly housed under standard lighting (14:10-hour light/dark cycle), humidity (50–60%) and temperature (22–23°C) conditions. Food and water were available ad libitum.

### Voluntary Exercise Paradigm

After habituation to the housing conditions for 5 days, the experimental group was allowed free access to a running wheel (diameter 34 cm) in their home cages for a period of four weeks. The rats ran approximately 4–7 km per night which is in agreement with other reports [13], [26]. The housing of sedentary (i.e. control) animals remained unchanged. All animal experiments were approved by the UK Home Office. Voluntary wheel running is not regarded as a form of stereotypic behavior [27] because, unlike other reported locomotor stereotypes, it is not expressed at the cost of resting behavior such as sleep [14] as is the case in other reported locomotor stereotypies [28], [29].

All experiments were carried out four weeks after voluntary exercise (or non-exercise) and between 8:00 and 12:00 h. For killing, individual rats were quickly anaesthetized (<15 sec) in a glass jar containing isoflurane (Merial Animal Health Ltd., UK) vapor, after which animals were decapitated immediately and their whole brains removed, snap frozen in isopentane at −40°C and deep-frozen in dry ice. Brains were stored at −80°C.

### Novel Environment Exposure

To induce novelty stress, as reported before [23], [30], [31] rats were placed singly for 30 min in a new cage (i.e. a clean cage with new sawdust but no food or water) in a separate room with identical environmental conditions except for increased light intensity (500 lx, holding conditions:100 lx). Behavior of rats was recorded using digital cameras and a hard disk recorder and later scored every 10 sec throughout the total 30 min duration of the test. The following behaviors were scored: lying (includes sleeping), rearing, stationary (standing or sitting), walking, grooming, scratching and burrowing behavior. Thereafter, rats were returned to their home cages and then placed in a recovery room (i.e. a room with identical environment and light conditions as the original holding room) until they were killed at 2 h after the onset of the novelty challenge.

### Forced Swimming

For forced swimming, as reported before [22], [24], [32], [33] rats were placed in a glass beaker (height 35 cm, diameter 21.7 cm) containing 25°C water (depth of 21 cm) for 15 min. Thereafter, the animals were dried with a towel and returned to their home cages and placed in a recovery room (see above) until they were killed at 2 h after the start of the forced swimming procedure. For both forced swimming and novelty stress, the 2 h time point was chosen as it has been described previously as the peak of P(Ser10)-Ac(Lys14)-H3 and c-Fos expression [23], [24].

For determination of forced swimming-induced acquisition of behavioral immobility, separate groups of rats were forced to swim for 15 min (as described above) and 24 h later were subjected again to forced swimming, in this case for 5 min (i.e. the ‘re-test’; water at 25°C). Behavioral immobility (or floating) is a behavioral state in which the animal retains an immobile posture displaying only enough movement to keep the head above water. Behavior in the initial test and re-test was recorded as described above and scored at a later time point. Three distinct behaviors were scored [immobility, struggling (also called climbing, in which the animal makes vertical movements along the wall of the beaker) and swimming (horizontal movements in the water)] every 10 s for the entire duration of the test and the re-test. In all cases scoring was conducted in a blinded fashion.

### Immunohistochemistry

Brain tissues were cut into coronal sections using a cryostat and mounted on glass slides (Superfrost, Fisher, Loughborough, UK) previously coated with poly-L-lysine (Sigma). Sections of rat brain were taken from the nucleus accumbens, PVN and the dorsal hippocampus in accordance with the atlas of Paxinos and Watson (1986) (AP co-ordinates: between AP 1.60 mm and 1.00 mm from Bregma for nucleus accumbens; between −1.80 mm and −2.12 mm from Bregma for PVN; between −2.92 mm and −3.96 mm from Bregma for the dorsal hippocampus). Sections were stored at −20°C until use. Storage at this temperature does not affect levels of P(Ser10)-Ac(Lys14)-H3 or c-fos in sections.

Immunohistochemical staining using diaminobenzidine (DAB) was conducted according to standard protocol as previously described [23], [24], [34] In brief, the brain sections were fixed in 4% paraformaldehyde in 1× phosphate-buffered saline (PBS) for 30 min. Thereafter, endogenous peroxidase activity was blocked by a 30-min incubation in 0.6% H_2_O_2_. To improve antibody penetration of the tissue, the sections were incubated for 1 h in 0.2% Triton X-100, followed by blocking of sections with 5% goat serum in PBS to prevent non-specific binding. The primary antibodies were diluted in 1.5% goat serum/PBS. Rabbit polyclonal antibody against P(Ser10)-Ac(Lys14)-H3 (dilution 1∶1,000) was purchased from Upstate (Charlottesville, VA, USA) and the rabbit anti-c-Fos antibody (used dilution 1∶10,000) was purchased from Calbiochem (Nottingham, UK). Incubation with primary antibodies occurred overnight at room temperature.

Biotinylated secondary antibody, avidin-biotin-peroxidase complex and DAB/Ni^+^ substrate (Elite-ABC and DAB detection kits; Vector Laboratories, Burlingame, CA, USA) for the development of immunostaining were used according to company instructions. After dehydration in ethanol, sections were finally mounted using Histomount (Fisher) and coverslipped.

### Data Analysis

The numbers of both P(Ser10)-Ac(Lys14)-H3^+^ neurons and c-Fos-positive (c-Fos^+^) neurons in the dentate gyrus (six sections per animal) were counted by an individual blind to the treatment. The location of each positive neuron was distinguished between the dorsal and ventral blade of the dentate gyrus. For analysis of the nucleus accumbens, two sample areas dorso-lateral to the anterior commissure were used to count positive neurons. For each antibody and brain region at least 2 assays were performed. The assays provided similar results and data of one assay is presented here. The experimental data were statistically evaluated using ANOVA and, if significant, followed by the post-hoc Bonferroni test as appropriate. Behavioral data of the novelty challenge were statistically tested using ANOVA with repeated measures followed by Student's t-test in appropriate cases. Forced swim test data were evaluated using Student's t-test. The experimental data were considered to be statistically different from control data when P<0.05.

## Results

### Novel environment-induced changes in P(Ser10)-Ac(Lys14)-H3^+^ and c-Fos^+^ neurons in the dentate gyrus of exercised and control rats

**Figure 1:**
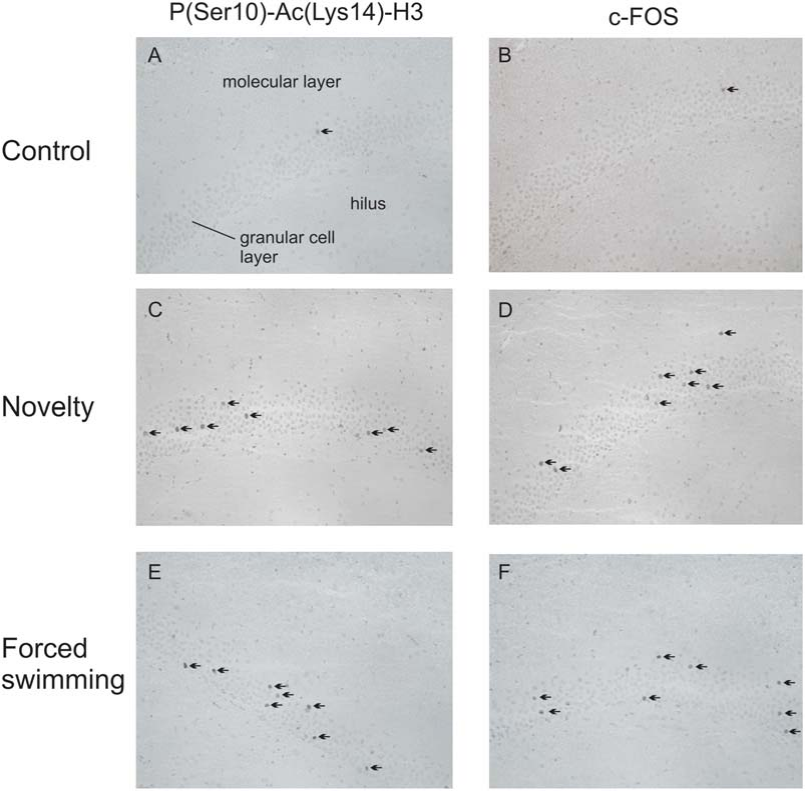
Representative images of anti-P(Ser10)-Ac(Lys14) (left panels) and anti-c-Fos (right panels) immuno-staining in the dorsal blade of the dentate gyrus of control rats under baseline conditions or at 2 h after novelty exposure or forced swimming. Black arrows indicate positive nuclear immuno-staining. Immunohistochemistry and the challenge tests were conducted as described in the Materials and Methods.

We first examined whether, in terms of histone H3 phospho-acetylation and c-Fos induction, exercised rats would respond differently than sedentary control animals to exposure to a mild psychological challenge such as a novel environment. Figure 1A–D shows representative immunohistochemical images of the dorsal blade of the dentate gyrus of control rats killed under baseline conditions or 2 h after novelty stress. Nuclear staining of phospho-acetylated histone H3 immunoreactivity can be seen in distinct granule neurons but no immunostaining was observed in hippocampal pyramidal neurons except for very few CA3 pyramidal neurons (data not shown). As reported before [22]–[24], only few P(Ser10)-Ac(Lys14)-H3^+^ neurons were found elsewhere in the brain. As previous *in vitro* [19] and *in vivo* [23], [24] research has shown that histone H3 phospho-acetylation is associated with the induction of immediate-early gene products such as c-Fos, we investigated whether exercised rats showed distinct responses in this gene product to novelty stress as well. The staining pattern for both P(Ser10)-Ac(Lys14)-H3^+^ and c-Fos^+^ neurons in the dentate gyrus was sparse which concurs with previous observations [22]–[24]. Novelty stress resulted in an increase in the number of both P(Ser10)-Ac(Lys14)-H3^+^ and c-Fos^+^ neurons (Fig. 1C, D).

**Figure 2:**
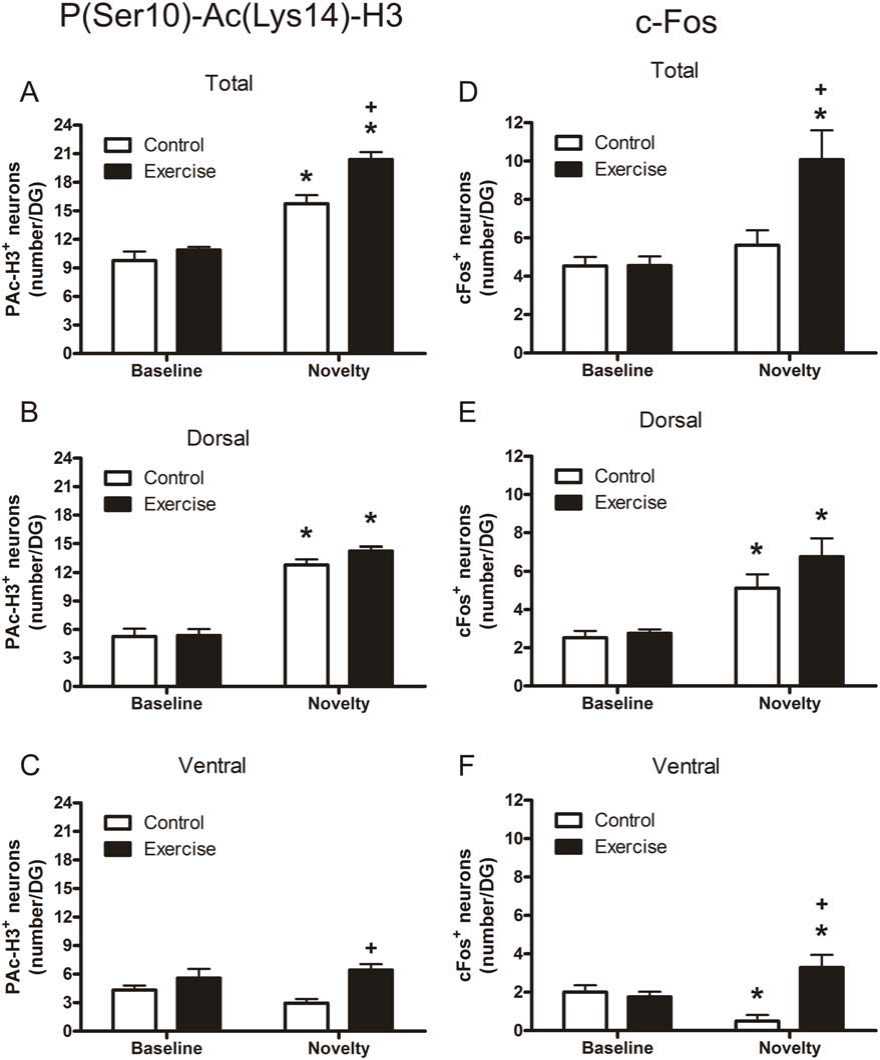
Effect of novelty exposure on the number of P(Ser10)-Ac(Lys14)^+^ (Left panels A, B, and C) and c-Fos^+^ neurons (right panels D, E and F) in the dentate gyrus of control, sedentary and 4-weeks exercised rats. A and D show data on total number of immuno-positive neurons in the dentate gyrus whereas in B and E and in C and F data are depicted separately for the dorsal blade and the ventral blade, respectively. Rats were allowed to voluntarily exercise by giving them access to a running wheel in their home cage. Data are expressed as the number of immuno-positive neurons (mean±SEM, n = 6) in the dentate gyrus of a 10-µm section. For additional information, see Materials and Methods. Statistical analyses: Two-way ANOVA: A, Effect of exercise: F(1,20) = 12.823, P = 0.002, Effect of novelty: F(1,20) = 93.616, P<0.0005, Interaction exercise x novelty: F(1,20) = 4.808, P = 0.043; B, Effect of Novelty: F(1,20) = 163.33, P<0.0005; C, Effect of exercise: F(1,20) = 13.346, P = 0.002; D, Effect of exercise: F(1,20) = 7.392, P = 0.013, Effect of novelty: F(1,20) = 15.921, P = 0.001, Interaction exercise x novelty: F(1,20) = 7.203, P = 0.014; E, Effect of novelty: F(1,20) = 33.302, P<0.0005; F, Effect of exercise: F(1,20) = 11.827, P = 0.003, interaction exercise x novelty: F(1,20) = 16.875, P = 0.001. *, P<0.05, compared to the respective Baseline group; ^+^, P<0.05, compared to the respective Control group, post-hoc Bonferroni test.

In terms of P(Ser10)-Ac(Lys14)-H3***^+^*** neurons in the whole dentate gyrus, novel environment exposure resulted in a significant increase in both the control and exercised animals, but the increase in exercised rats was substantially greater than that in the control animals (Fig. 2A). If the dorsal and ventral blade of the dentate gyrus were considered separately, a different picture emerged. Considering the dorsal blade separately, the response in P(Ser10)-Ac(Lys14)-H3***^+^*** neurons to a novelty challenge was similar in exercised and control animals (Fig. 2B). However, analysis of the ventral blade showed that novelty stress resulted in higher numbers of P(Ser10)-Ac(Lys14)-H3***^+^*** neurons in exercised animals than in control animals (Fig. 2C). Apparently, these higher numbers in the ventral blade of exercised rats were the principal reason for the enhanced histone H3 phospho-acetylation response observed in the whole dentate gyrus after novelty stress. In line with previous findings [23], the P(Ser10)-Ac(Lys14)-H3***^+^*** neurons were mainly found in the middle and superficial aspects of the granular cell layer, with an overall greater abundance in the dorsal blade (data not shown).

With regard to c-Fos, we found that considering the whole dentate gyrus novelty stress only evoked a significant increase in c-Fos***^+^*** neurons in the dentate gyrus of exercised animals (Fig. 2D). Surprisingly, control animals did not show a significant increase over baseline levels when total numbers in the dentate gyrus were considered. However, when considering the dorsal and ventral blade separately and comparing values to those observed under baseline conditions, novelty stress evoked an increase in cFos***^+^*** neurons in the dorsal blade whereas decreased numbers were observed in the ventral blade (Fig. 2E, F). A more uniform response was observed in the exercised rats where the same stressor caused an increase in the number of cFos***^+^*** neurons in the dorsal blade as well as in the ventral blade (Fig. 2E, F).

### Novel environment-induced changes in behavior of exercising animals

**Figure 3:**
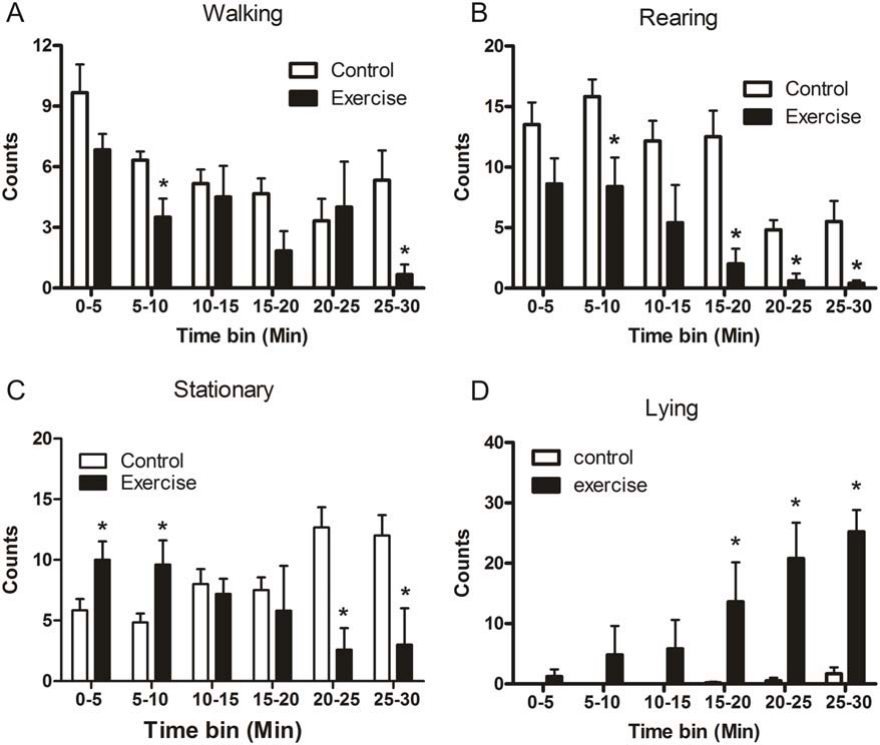
Behavior of control and exercised rats during exposure to a novel environment, i.e. a new cage in a brightly lit (500 lx) room. Changes in walking (A), rearing (B), stationary (C) and lying behavior (D) were scored every 10 sec throughout the 30-min novelty exposure. Data were binned in 5-min time bins and expressed as behavioral counts (mean±SEM, n = 6). Statistical analyses: Two-way ANOVA with repeated measures: A, Effect of time: F(5,45) = 5.387, P = 0.001, Effect of exercise: (F1,9) = 4.739, P = 0.057, Interaction time x exercise: F(5,45) = 1.331, not significant; B, Effect of time: F(5,45) = 15.545, P<0.0005, Effect of exercise: F(1,9) = 14.263, P = 0.004, Interaction time x exercise: F(5,45) = 1.305, not significant; C, Effect of time: F(5,45) = 0.153, not significant, Effect of exercise: F(1,9) = 1.878, not significant, Interaction time x exercise: F(5,45) = 7.881, P<0.0005; D, Effect of time: F(5,45) = 11.529, P<0.0005, Effect of exercise: F(1,9) = 11.332, P = 0.008, Interaction time x exercise: F(5,45) = 9.130, P<0.0005. *, P<0.05, Student's t-test.

To assess whether differential behavioral coping strategies are involved in the distinct histone modification and gene expression responses in exercised and control rats, we scored various behaviors displayed by the animals in the novel environment (Fig. 3). Rats showed mainly exploratory behaviors such as walking (Fig. 3A), rearing (Fig. 3B) and burrowing (data not shown) when introduced in the novel cage. There were no differences between the experimental groups at this early stage. The exercising rats showed more stationary behavior during the first 10 min in the novel cage (Fig. 3C). This observation corresponds with our earlier observations in exercised mice. When placed in an open field these animals also initially show more stationary behavior as a result of increased vigilance and decreased impulsiveness [15].

Over the course of time in the novel cage the exercised rats showed significantly less walking and rearing behavior suggesting a gradual decline in exploratory behavior in these animals (Fig. 3A, B). Indeed, the exercised rats lay down much more than the control animals during the second half of the novel cage test (Fig. 3D). It appeared that some animals slept whilst lying down but this cannot be assessed with certainty as sleep/EEG measurements were not conducted. The control rats maintained walking and stationary, and to some extent, rearing behavior throughout and until the end of the novel cage test. Rats did not show differences in grooming, scratching and burrowing behavior (data not shown). Thus, whereas the control rats kept exploring the novel environment for the complete exposure time, the exercised animals appeared to lose interest over time and returned to their normal behavior at that time of day, being resting or sleeping.

### Forced swimming-induced changes in P(Ser10)-Ac(Lys14)-H3^+^ and c-Fos^+^ neurons in the dentate gyrus

In contrast to the novelty stress paradigm which largely allows passive coping strategies, forced swimming is a challenge that enforces active coping styles such as struggling and swimming as well as adaptive coping strategies such as immobility or floating behavior. Furthermore, whereas the novelty stress paradigm only addresses acute behavioral responses to the situation, the forced swim paradigm includes behavioral responses to the acute situation (i.e. the initial forced swim test) as well as behavioral responses upon re-exposure to the forced swim challenge (i.e. the re-test). Previously, we have shown that histone H3 phospho-acetylation and c-Fos induction in dentate granule neurons is required for the acquisition of immobility behavior seen in the re-test [22], [24].

**Figure 4:**
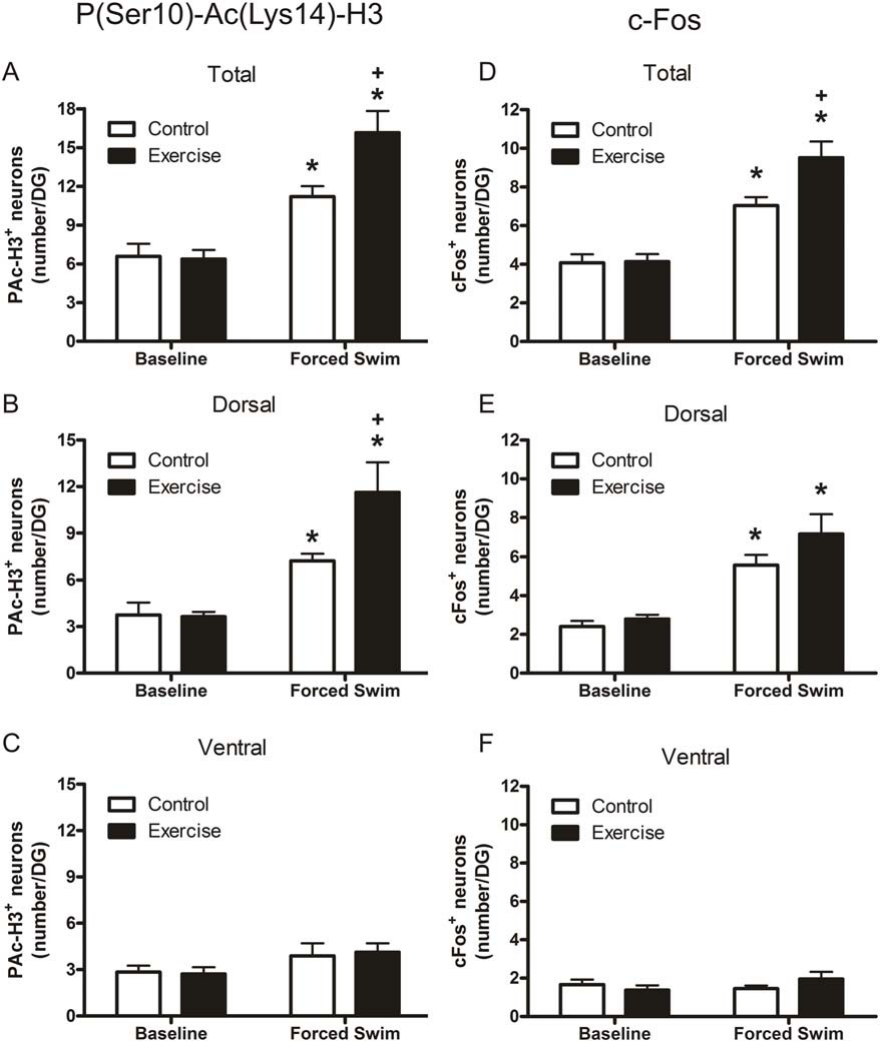
Effect of forced swimming on the number of P(Ser10)-Ac(Lys14)^+^ (Left panels A, B, and C) and c-Fos^+^ neurons (right panels D, E and F) in the dentate gyrus of control, sedentary and 4-weeks exercised rats. A and D show data on total number of immuno-positive neurons in the dentate gyrus whereas in B and E and in C and F data are depicted separately for the dorsal blade and the ventral blade, respectively. Data are expressed as the number of immuno-positive neurons (mean±SEM, n = 6) in the dentate gyrus of a 10-µm section. For additional information, see Materials and Methods. Statistical analyses: Two-way ANOVA: A, Effect of exercise: F(1,24) = 3.495, P = 0.077, Effect of forced swimming: F(1,24) = 32.292, P<0.0005, Interaction exercise x forced swimming: F(1,24) = 4.135, P = 0.056; B, Effect of exercise: F(1,24) = 21.144, P<0.0005, Interaction exercise x forced swimming: F(1,24) = 3.257, P = 0.087; D, Effect of exercise: F(1,24) = 5.598, P = 0.026, Effect of forced swimming: F(1,24) = 59.533, P<0.0005, Interaction exercise x forced swimming: F(1,24) = 4.993, P = 0.035; E, Effect of forced swimming: F(1,24) = 38.318, P<0.0005. *, P<0.05, compared to the respective Baseline group; ^+^, P<0.05, compared to the respective Control group, post-hoc Bonferroni test.

Figure 1 shows representative immunohistochemical images of the dorsal blade of the dentate gyrus of rats killed under baseline conditions or 2 h after forced swimming. Forced swimming indeed evoked an increase in histone H3 phospho-acetylation and c-Fos in dentate granule neurons as compared to control animals (Fig. 1A, B, E, F). Furthermore, counting of the immuno-stained neurons revealed that the increase in the number of P(Ser10)-Ac(Lys14)-H3***^+^*** neurons after forced swimming was significantly higher in the exercised rats than in the control animals (Fig. 4). Separate analyses of the dorsal and ventral blades showed that the forced swimming-induced response was confined to the dorsal blade, where there were almost twice as many (Ser10)-Ac(Lys14)-H3***^+^*** neurons in the exercised animals than in the controls (Fig. 4B). There was no effect of forced swimming on the number of P(Ser10)-Ac(Lys14)-H3***^+^*** neurons in the ventral blade (Fig. 4C).

Analysis of c-Fos immunostaining demonstrated a pattern of forced swimming-induced changes in control and exercised rats that was largely similar to that found for P(Ser10)-Ac(Lys14)-H3, at least if the whole dentate gyrus was considered (Fig. 4D). Thus, forced swimming resulted in enhanced c-Fos expression in the dentate gyrus of both control and exercised animals but the response in the exercised group was significantly higher. However, in the dorsal blade and in contrast to the P(Ser10)-Ac(Lys14)-H3 data, this enhanced response was not as apparent because the difference between the stressed control and stressed exercised animals only showed a trend (Fig. 4E). The c-Fos data for the ventral blade were parallel to those found for the P(Ser10)-Ac(Lys14)-H3 data (Fig. 4F).

### Exercised rats show improved adaptive behavior after forced swimming

**Figure 5:**
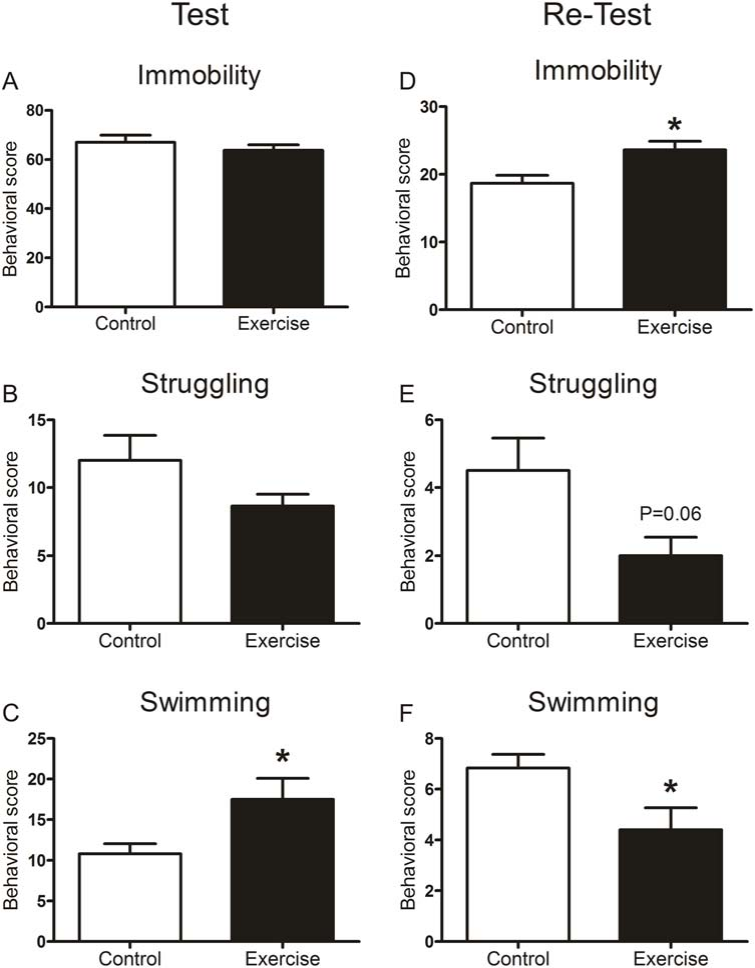
Behavior of control and exercised rats in the forced swim test. Rats were subjected to an initial test of 15 min in 25°C-water followed by a 5-min re-test 24 h later. Immobility, struggling and swimming behavior during the test (left panels) and re-test (right panels) was scored every 10 sec. Data are expressed as the accumulated behavioral scores (mean±SEM, n = 6). *, P<0.05, Student's t-test.

Behavior of rats during the initial forced swim test and the re-test were recorded and scored to assess whether changes in histone H3 phospho-acetylation and c-Fos expression in the exercised rats were related to behavioral changes in this paradigm. In the initial forced swim test, control and exercised rats showed largely similar behaviors (Fig. 5A–C), with similar levels of immobility and struggling behavior but slightly more swimming behavior in the exercised animals than in the controls. However, in the retest 24 h later, the exercised rats showed significantly higher immobility and lower swimming and struggling scores than the controls (Fig. 5D–F). Given that the immobility response in the re-test is regarded as a reflection of the strength of the memories formed after the initial forced swim event, the enhanced immobility response in the exercised rats suggests that these animals have improved cognitive and adaptive abilities to cope with psychologically stressful events.

### Forced Swimming-induced changes in cFos^+^ neurons in the nucleus accumbens

**Figure 6:**
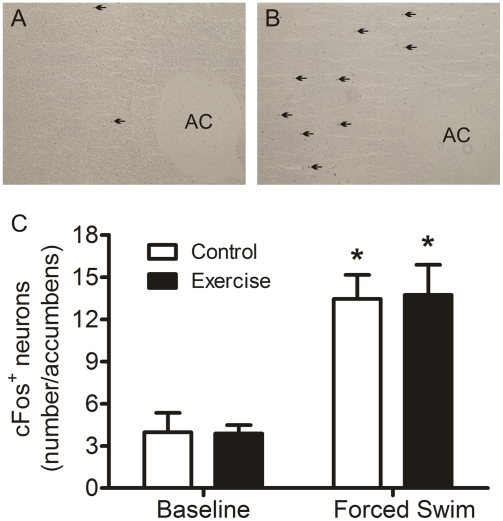
Effect of forced swimming on c-Fos expression in the nucleus accumbens of control and exercised rats. A and B show representative images of anti-c-Fos immuno-staining in an area of the nucleus accumbens dorso-lateral to the anterior commissure (AC). This area comprises parts of both the core and shell regions. Black arrows indicate positive nuclear immuno-staining. C shows the number of c-Fos^+^ neurons in this area of control and exercised rats under baseline conditions and at 2 h after forced swimming. Statistical analysis: Two-way ANOVA: Effect of forced swimming: F(1,16) = 38.157, P<0.0005. *, P<0.05, compared to the respective Baseline group, post-hoc Bonferroni test.

The nucleus accumbens is a mesolimbic brain region involved in behavioral responses to stress. It has also been suggested to act as a neuroanatomical substrate for immobility/floating behavior in the forced swim test [35]. Therefore, we investigated whether forced swimming would lead to a differential c-Fos induction in the nucleus accumbens of exercised rats as compared to control animals. These animals were the same as those used for the dentate gyrus analyses. Figure 6A and B show representative images of the nucleus accumbens of rats killed under baseline conditions or at 2 h after forced swimming. We counted c-Fos^+^ neurons in an area of the nucleus accumbens showing highest numbers of immuno-positive neurons, comprising parts of both the core and shell region. Forced swimming induced a marked increase in the number of c-Fos^+^ neurons in the nucleus accumbens of both control and exercised animals. The increase, however, was similar in both groups (Fig. 6C). The similar degree of c-Fos induction in exercised and control animals suggests that any differential behavioral responses to stress in the exercisers are unlikely to be mediated by the nucleus accumbens.

We also analyzed the hypothalamic paraventricular nucleus (PVN), a stress-sensitive nucleus that plays a principal role in hypothalamic-pituitary-adrenocortical (HPA) axis regulation [17]. We found that the increases in c-Fos levels were similar in control and exercised rats after forced swimming (data not shown).

## Discussion

In the present study, we show that exercised rats present improved coping responses and memory performance after exposure to a novel environment or a forced swim test. Furthermore, these behavioral changes were associated with enhanced responses in histone H3 phospho-acetylation and c-Fos in dentate gyrus granule neurons. In contrast, the immediate early gene responses in the nucleus accumbens and PVN were similar in exercised and sedentary control animals. Therefore, it appears that changes in histone H3 phospho-acetylation and gene expression responses in the dentate gyrus are involved in the enhanced stress-coping capabilities seen in exercised animals.

Exercised rats adopted a different coping strategy than control animals when faced with the novelty challenge. They exhibited less emotionality than their non-running counterparts, exploring their new environment initially but then settling down. In contrast, the non-running, sedentary rats remained active virtually for the full 30 min. These findings correspond with our previous work in which we showed that regular voluntary exercise reduces anxiety-related behavior and novelty-induced glucocorticoid responses [11], [13], [15]. Thus, exercised animals cope better with this mild psychological challenge situation. As they basically stopped exploring the novel environment after 15 min and returned to their normal behavior of this time of the day (which is resting or sleeping), it seems that exercised animals are much quicker than their sedentary counterparts in assessing the novel situation. This may relate to the enhanced cognitive abilities reported in exercised animals [36].

Although the novelty situation appeared to have less influence on the exercised rats, they showed significantly higher dentate histone H3 phospho-acetylation and c-Fos responses than the control animals. In both groups of rats the challenge mainly impacted on the dorsal (or suprapyramidal) blade of the dentate gyrus which is in agreement with our previous results [23]. Currently, very little is known with regard to neuroanatomical and functional differences between the dorsal and the ventral (infrapyramidal) blade. The dentate gyrus receives its major afferent input from Layer II of the lateral and medial entorhinal cortex but these afferents seem to be distributed equally in density between the dorsal and the ventral blade [37]. At the receiving side, however, it appears that the granule neurons of the dorsal blade show more extensive arborizations than those of the ventral blade. Regarding subcortical regions, the dentate gyrus receives input from the septal nuclei, the locus coeruleus, the supramammillary area and the raphe nuclei [37]–[39]. The supramammillary area is of special interest, as the dorsal blade receives double as many fibres from this area than the ventral blade [40] and is implicated in hippocampus-regulated emotional and cognitive functions [39], [41]. Interestingly, the ventral blade of the exercised rats showed a relatively higher response than that of the control animals which may indicate an enhanced engagement of this part of the dentate in the response to novelty after long-term exercise. The reason for this enhanced participation remains presently unclear.

In contrast to the novelty challenge test, the forced swim test is a test to address changes in active coping styles such as struggling and swimming as well as adaptive coping strategies such as immobility or floating behavior. Apart from the moderate increase in swimming behavior during the initial test in exercised rats, there were no significant differences between the control and exercised animals. Nevertheless, there were substantial differences regarding the impact of the challenge on dentate gyrus granule neurons. After forced swimming, the exercised rats, as compared to the sedentary animals, showed a significantly higher number of P(Ser10)-Ac(Lys14)-H3^+^ and c-Fos^+^ neurons in the dentate gyrus. In conjunction with observations in the novelty paradigm, it seems that the neuronal response does not directly relate to the immediate behavioral reaction *during* the challenge but rather relates to how the information is being processed by the dentate gyrus in the hours *after* the challenge. The difference in processing materialized the next day when animals were re-exposed to the forced swim test. At this time, the exercised rats showed more pronounced immobility behavior than the sedentary controls. We reported before that the phospho-acetylation of histone H3 and c-Fos induction in dentate granule cells seen after the initial forced swim test is strongly associated with the immobility behavior response observed 24 h later in the re-test [22], [24]. This behavioral response is increasingly regarded as a reflection of the strength of memory of the first forced swim experience [21], [42], [43]. Moreover, we recently stipulated that these mechanisms may also play a role in the formation of traumatic, pathological memories as occurring in post-traumatic stress disorder (PTSD; [25]). We found that any interruption (due to pharmacological intervention or gene deletion) of the signaling cascade initiating the histone modifications and immediate-early gene induction in dentate granule neurons resulted in an impaired immobility response [22], [24]. Earlier work provided evidence specifically pointing to a critical role of the glucocorticoid receptor located in the dentate gyrus in the forced swimming-induced immobility response [44]. There have been reports about a role of the nucleus accumbens in immobility behavior. This nucleus has been studied in relation to immobility behavior interpreting this behavior as being an indicator of learned helplessness or depressive behavior [35], [45]. However, our data question this interpretation because our exercised animals showed increased immobility behavior in the re-test. Interpretation of this increased immobility as indicating increased depressive behavior would be highly debatable given that exercised animals are known to be less anxious and cognitively better than sedentary control animals [15], [36]. Evidence is accumulating that exercise is anxiolytic and antidepressant in humans [7]–[10]. Furthermore, since exercised and control rats produced similar c-Fos responses in the nucleus accumbens in the face of different immobility responses in the re-test, it seems that this brain structure does not play a critical role in the differential immobility responses in exercised and control rats. Moreover, mitogen and stress-activated kinase 1/2 (MSK1/2) double knockout mice showed highly impaired immobility behavior in the re-test of the forced swim test in conjunction with virtually absent histone H3 phospho-acetylation and c-Fos responses to forced swimming in the dentate gyrus; elsewhere in the brain (including the nucleus accumbens) c-Fos responses to forced swimming were normal [24](Chandramohan Y and Reul JMHM, unpublished observations). Collectively, these results point to behavioral immobility reflecting an adaptive response in which formation of memories of the initial swim event plays an important role. In addition, these cognitive processes seem to involve distinct epigenetic and gene expression mechanisms in dentate granule neurons.

Our previous work has shown that the phospho-acetylation of histone H3 in dentate neurons after forced swimming and novelty is brought about by concurrent signaling via the GR and NMDA/ERK/MSK pathways [21]–[25]. Currently the mechanisms underlying the enhanced epigenetic and gene expression responses in the exercised animals are unknown. Yet, a variety of possible mechanisms contributing to the altered responses after exercise can be identified. Changes in the two principal pathways identified by us, i.e. NMDA receptors and GRs, may be involved. Recent work has shown that exercise leads to changes in NMDA receptor composition and NMDA receptor-related neuroplasticity processes [46]–[48]. Furthermore, we reported recently that the expression of GRs is increased in the hippocampus of exercised rats resulting most likely in an enhanced impact of stress-induced elevations in glucocorticoid hormone levels [13], [49]. Other neurotransmitter systems possibly involved in the differential epigenetic and gene expression responses in the exercised rats include the central noradrenergic, serotonergic and GABAergic systems. These systems are known to modulate dentate neuron excitability and are known to be altered after voluntary exercise or during general motor activity [11], [33], [50]–[52] (Papadopoulos A., Chandramohan Y., Collins A., Droste S.K., Nutt D.J. and Reul J.M.H.M., unpublished observations). In addition, changes in intracellular pathways such as the ERK/MSK pathway and/or histone acetyl transferase (HAT) activities cannot be excluded. Finally, it also cannot be ruled out that changes in the cyto-architecture of the dentate gyrus may have contributed to the enhanced responses in histone H3 phospho-acetylation and c-Fos expression to forced swimming and novelty in the exercised rats.

Here we showed that exercised rats have improved capabilities to cope with psychologically stressful challenges. This enhanced stress coping materialized during the exposure to a novel environment and when re-submitted to a forced swim challenge. This improved adaptive capacity may be the logical consequence of the complex of elevated cognitive abilities, lowered anxiety levels and decreased impulsiveness known of exercised subjects [13], [15], [36]. The increased responses in histone H3 phospho-acetylation and c-Fos induction in dentate granule neurons of exercised rats strengthens our concept that these epigenetic and gene expression responses are part of neuroplasticity processes in the hippocampus aimed at establishing memories of the event in case the event would re-occur in the future. Further investigation of these mechanisms should be of great relevance for the elucidation of stress-related psychiatric disorders such as major depression and PTSD.
